# Use of activated recombinant factor VII for severe coagulopathy post ventricular assist device or orthotopic heart transplant

**DOI:** 10.1186/1749-8090-2-32

**Published:** 2007-07-06

**Authors:** Manish J Gandhi, Richard A Pierce, Lini Zhang, Marc R Moon, George J Despotis, Nader Moazami

**Affiliations:** 1 Department of Pathology and Immunology, Washington University School of Medicine, 660 S Euclid Ave, St Louis, MO 63110, USA; 2 Department of Surgery, Division of Cardiothoracic Surgery, Washington University School of Medicine, 660 S Euclid Ave, St Louis, MO 63110, USA; 3 Department of Anesthesiology, Washington University School of Medicine, 660 S Euclid Ave, St Louis, MO 63110, USA; 4 Mayo Clinic, Division of Transfusion Medicine, 200 First St SW, Rochester, MN 55901

## Abstract

**Background:**

Ventricular assist devices(VAD) implantation/removal is a complex surgical procedure with perioperative bleeding complications occurring in nearly half of the cases. Recombinant activated factor VII (rFVIIa) has been used off-label to control severe hemorrhage in surgery and trauma. We report here our experience with rFVIIa as a rescue therapy to achieve hemostasis in patients undergoing orthotopic heart transplant (OHT) and/or VAD implantation.

**Methods:**

A retrospective review was conducted from Jan 03 to Aug 05 for patients who received rFVIIa for the management of intractable bleeding unresponsive to standard hemostatic blood component therapy. Blood loss and the quantity of blood products, prior to, and for at least 12 hours after, administration of rFVIIa were recorded.

**Results:**

Mean patient age was 53, (38–64 yrs), mean dose of rFVIIa administered was 78.3 μg/kg (24–189 μg/kg) in 1–3 doses. All patients received the drug either intraoperatively or within 6 hours of arrival in ICU. Mean transfusion requirements and blood loss were significantly reduced after rFVIIa administration (PRBC's; 16.9 ± 13.3 to 7.1 ± 6.9 units, FFP; 13.1 ± 8.2 to 4.1 ± 4.9 units, platelets; 4.0 ± 2.8 to 2.1 ± 2.2 units, p < 0.04 for all). 5 patients expired including 3 with thromboembolic cause. One patient developed a lower extremity arterial thrombus, and another deep vein thrombosis.

**Conclusion:**

In this review, there was a significant decrease in transfusion requirement and blood loss after rFVIIa administration. Although, 5/17 developed thromboembolic complications, these patients may have been at higher risk based on the multiple modality therapy used to manage intractable bleeding. Nevertheless, the exact role of rFVIIa with respect to development of thromboembolic complications cannot be clearly determined. Further investigation is needed to determine rFVIIa's safety and its effectiveness in improving postoperative morbidity and mortality.

## Background

Ventricular assist devices (VADs) have been in widespread clinical use for long-term support of patients with end-stage heart failure as a bridge to transplantation. These devices are compatible with low long term morbidity, hemodynamic stability along with significant metabolic and physical rehabilitation. However, VAD implantation and explantation are complex surgical procedures with perioperative bleeding occurring in nearly half of the cases [1,2]. The amount of blood and blood products used during these operations is substantial, and cardiac surgery including VAD implantation/explantation now account for approximately 15% of the yearly utilization of the entire banked blood supply in both the US and the UK [3,4]. In addition to issues related to cost and availability of blood and blood products, large volume blood product transfusions are associated with the risk for transfusion-related reactions, infectious complications, increased pulmonary vascular resistance, possible alloreactivity against transplanted organs, and even lower long-term survival well outside the perioperative period [5].

Further in patients undergoing complex cardiac surgeries requiring re-explorations [6-9], only 50% of the patients had a surgical source of bleeding which demonstrates the important role of acquired hemostatic abnormalities that result in diffuse, microvascular bleeding. In addition, the analysis by Moulton et al demonstrated that excessive bleeding is the most likely cause of increased mortality since mortality was also increased by three-fold when patients who were not re-explored bled more than two liters within the first 24 hours after surgery [6]. Excessive bleeding requiring transfusion may also result in other complications such as stroke and may affect long term mortality. In a large, recently published analysis, excessive bleeding requiring more than four units of PRBC transfusion was the strongest (odds ratio = 5) independent predictor with respect to perioperative stroke [10]. In yet another study, massive blood loss (receiving at least five units of PRBC within 24 hours of surgery) in cardiac surgery was associated with an 8.1-fold (95% confidence interval, 3.9–17.0) increase in the odds of death [9].

Recombinant Factor VIIa (rFVIIa, NovoSeven^®^, Novo Nordisk Inc, Princeton, NJ) is a potent clotting factor currently FDA-approved only for the control of bleeding in hemophiliacs with factor VIII (FVIII) or factor IX (FIX) inhibitors or patients with Factor VII deficiency. rFVIIa is increasingly being used off-label for control of coagulopathic bleeding unresponsive to conventional measures in cardiac surgical patients [3,4,11-15]. In our retrospective case series, we report our experience with the use of rFVIIa in the treatment of refractory bleeding in patients with VAD implantation or explantation and orthotopic heart transplant (OHT).

## Methods

Following institutional review board approval, we conducted a retrospective chart review from January 2003 to August 2005 involving patients who had either VAD implantation or explantation and OHT. At our institution cardio-pulmonary bypass (CPB) was performed using a non-coated circuit and a membrane oxygenator. Anticoagulation was generally achieved with unfractionated heparin and anticoagulation was monitored with kaolin activated clotting time (ACT) and whole blood heparin levels (i.e., via Hepcon automated protamine titration method, Medtronic, Minneapolis, MN) every 30 minutes with a target of ≥ 480 seconds and/or a whole blood heparin level ≥ than the baseline level associated with a therapeutic ACT prior to initiation of CPB as previously described [16]. Only one patient with a history of recent HIT underwent CPB with bivalirudin. All patients without renal dysfunction received aprotinin (25,000 U/kg or 3.5 mg/kg for both the loading and CPB doses with 1/4 of that dose every hour by infusion) after heparinization. During CPB the hematocrit (Hct) was maintained between 0.21 to 0.25. After CPB, the cutoff values for red cell transfusion was Hct < 0.30, for fresh frozen plasma (FFP) and platelets were based on the initial platelet count, prothrombin time (PT) and activated partial thromboplastin time (aPTT) as previously described [17]. Patients who had continued bleeding and uncorrected coagulopathy after complete reversal of heparin with protamine and had received standard conventional component blood therapy were identified. Conventional component therapy was administered taking into account the effect of CPB on platelets and coagulation factors. Seventeen such patients were identified. The decision to give rFVIIa was based on individual physician discretion. In general, the decision to administer rFVIIa was based on continued bleeding related to coagulopathy with no identifiable surgical source. In all cases, patients had received hemostatic blood components in an attempt to reverse coagulopathy but bleeding persisted.

Patient characteristics, rFVIIa dose, clinical outcomes and complications including thrombotic episodes were noted. All blood loss prior to, and for at least 12 hours after, administration of rFVIIa was recorded. Complete blood counts and coagulation studies were obtained to assess for ongoing hemorrhage and/or coagulopathy. Furthermore, the amount of blood and hemostatic blood products each patient received were recorded both before and after receiving rFVIIa.

Complete data on blood product utilization and bleeding was available for 15 subjects who were included in the analysis. Mortality data was available for all 17 patients. During the study period a total of 200 such procedures (VADs:134 and OHT: 66) were performed at our institute. Student's t-test was used to compare values. Unless otherwise indicated, all values are represented as Mean ± Standard Deviation.

## Results

### Demographic and Surgical Data

The demographic and surgical data are illustrated in table 1. The mean age of the patients was 53 years (range; 38–67 years) and majority (73%) were male. All patients were Caucasians. None of the patients had a history of a pre-existing coagulopathy. Two patients had heparin-induced thrombocytopenia (HIT); one with a known history and another diagnosed subsequent to thrombocytopenia following the left VAD (LVAD) implantation. Majority of the patients (9/15) had removal of the bridging VAD followed by OHT. Five patients had VAD implantation (three as destination therapy, two for post-cardiotomy shock), while one patient had only orthotopic heart transplant (OHT). In one of the patients bridged to transplant with a LVAD, post transplant allograft failure necessitated placement on extracorporeal membrane oxygenation (ECMO) support.

**Table 1:** Patient demographics, rFVIIa dose, mortality and thrombo-embolic complications

**Pt**	**Sex**	**Age (yrs)**	**1^st ^Dose (mg)**	**2^nd ^Dose (mg)**	**3^rd ^Dose (mg)**	**Total rFVIIa (μg/kg)**	Procedure*	**Death**	**Cause of Death**	**TE episode**	**Associated Risk Factors**
1	M	51	7.2	4.8	-	143	VAD to OHT	Y	Graft Failure	N	N
2	M	53	2.4	-	-	24	VAD to OHT	N	-	N	N
3	M	42	2.4	-	-	36	VAD	N	-	Y (AT^Ψ^)	N
4	M	52	4.8	4.8	-	77	VAD to OHT	Y	CVA** (embolic)	Y	Atherosclerosis
5	M	61	2.4	1.2	-	36	VAD	N	-	N	HIT^
6	M	64	4.8	-	-	72	VAD to OHT	N	-	N	N
7	M	62	4.8	-	-	56	VAD to OHT	N	-	Y (DVT)^¥^	N
8	M	53	2.4	2.4	1.2	80	VAD	Y	CVA (air embolism)	?	N
9	M	57	2.4	2.4	1.2	71	OHT	N	-	N	N
10	M	53	4.8	-	-	48	VAD to OHT	N	-	N	N
11	F	38	1.2	1.2	-	32	VAD to OHT	N	-	N	N
12	F	51	2.4	6	6	189	VAD	Y	CVA (embolic)	Y	HIT^
13	F	67	4.8	2.4	-	109	VAD	Y	Heart Failure	N	Y (Multiorgan failure), ABIOMED^#^
14	F	46	4.8	4.8	1.2	150	VAD to OHT	N	-	N	N
15	M	49	4.8	-	-	49	VAD to OHT	N	-	N	N

*VAD = ventricular assist device, OHT = orthotopic heart transplant

**CVA = cerebro-vascular accident

^€^TE = thrombo-embolic

^Ψ^AT = Right lower extremity arterial thrombus

^¥^DVT = Deep vein thrombosis

^HIT = Heparin induced thrombocytopenia, ^#^ABIOMED = ABIOMED Left Ventricular Assist Device

### Recombinant Activated Factor VII dose

Complete data was available for 15/17 patients. Eight received rFVIIa intraoperatively, while seven received it within six hours of arriving in the cardiothoracic intensive care unit. The mean total dose of rFVIIa administered was 78.3 μg/kg (range, 24–189 μg/kg). All patients received at least one dose of rFVIIa with a maximum of three. There were no strict criteria regarding the dose of rFVIIa and the dose was based on consultation with transfusion medicine/hematology service and previously published guidelines [18]. Initial average rFVIIa dose administered was 40.1 μg/kg (range; 16–85.7 μg/kg). 9/15 patients were given a second dose for continued bleeding after the first dose. The average rFVIIa dose was 40.7 μg/kg (range; 12–79 μg/kg). Four patients continued to bleed after two doses and received a third dose; average 31.5 μg/kg (doses: 4.3 – 79 μg/kg). The patient that received the third dose of 79 μg/kg was the one who received the highest total dose of 189 μg/kg. This patient had HIT and developed a macrothrombi in the CPB circuit (within reservoir) during the conduct of the operation. The patient subsequently received multiple bolus doses of bivalirudin and post-operatively continued to bleed. None of the patients received rFVIIa prophylactically. Seven of the 15 patients (47%) required operative re-exploration for continued hemorrhage, but a surgical source of bleeding could be identified in only two of these patients.

### rFVIIa results in decreased blood loss and decreased transfusion requirement

Complete data was available for 15/17 patients. Blood loss was measured for each patient from the initiation of surgery and prior to, and for at least twelve hours after administration of rFVIIa. The blood loss prior to administration of rFVIIa was an average of 698 ± 285 ml/hr (median: 584 ml/hr). This blood loss dropped significantly to 186 ± 146 ml/hr (p = 0.001; median: 147 ml/hr) for the first six hours and 136 ± 127 ml/hr (p < 0.001; median: 108 ml/hr) for twelve hours immediately after administration of the drug (Table 2). The average pre- and post-treatment INR values were 2.34 ± 0.91 and 1.44 ± 0.93 (p = 0.001), with PTTs of 83.8 ± 43.4 and 53.7 ± 16.8 seconds (p = 0.030), respectively. The average hematocrit before (0.27 ± 0.79) and after rFVIIa administration were not significantly different (p = 0.175). Similarly the average platelet counts before (116 ± 71) and after (122 ± 51) rFVIIa administration were not significantly different (p = 0.379). This was likely secondary to ongoing resuscitative efforts.

**Table 2:** Blood loss, transfusion requirement, laboratory values prior to and following rFVIIa administration

	**Before rFVIIa Treatment**	**After rFVIIa Treatment**	**p Value**
Blood Loss (ml/hr)	698 ± 285	-	
6 Hour Blood Loss (ml/hr)	-	186 ± 146	0.001*
12 Hour Blood Loss (ml/hr)	-	136 ± 127	<0.001*
Packed Red Cells (Units)	16.9 ± 13.3	7.1 ± 6.9	0.037
Single Donor Platelets (Units)	4.0 ± 2.8	2.1 ± 2.2	0.024
FFP (Units)	13.1 ± 8.2	4.1 ± 4.9	<0.001
Cryoprecipitate (Units)	6.3 ± 6.3	0.3 ± 0.6	0.006
Hematocrit	0.27 ± 0. 79	0.29 ± 0.53	0.175
Platelet Count (x10^3^/dL)	116 ± 71	122 ± 51	0.379
PT (seconds)	21.4 ± 4.8	14.9 ± 3.5	<0.001
INR	2.34 ± 0.91	1.44 ± 0.93	0.001
PTT (seconds)	83.8 ± 43.4	53.7 ± 16.8	0.030

* p-value calculated as compared to blood loss before rVIIa administration

Mean transfusion requirements were significantly reduced, as patients received an average of 16.9 ± 13.3 (median: 14) units of packed red blood cells (PRBC), 13.1 ± 8.2 (median: 12) units fresh frozen plasma (FFP), 4.0 ± 2.8 (median: 3) units platelets and 6.3 ± 6.3 (median: 4) units cryoprecipitate before dosing but only 7.1 ± 6.9 (median: 7), 4.1 ± 4.9 (median: 2), 2.1 ± 2.2 (median: 1), and 0.3 ± 0.6 (median: 0) respective units of these blood products afterward (all p < 0.04) (table 2).

### Mortality and thrombotic complications

Mortality rate in this series of patients was 29% (5/17, table-2). One patient died from acute allograft failure that failed to recover with ECMO support. A second patient had undergone an aortic valve replacement at an outside institution and had been placed on an ABIOMED (ABIOMED, Danvers, MA) LVAD for temporary mechanical support and died from complications associated with multisystem organ failure. Three other patients died secondary to massive cerebrovascular accidents. One patient had HIT and was thus anticoagulated with bivalirudin on cardiopulmonary bypass. During the operation thrombus developed in the cardiopulmonary circuit and probably was the likely source for cerebral thromboembolism. Another patient was known to have atherosclerosis of the aorta and had also under gone a 35 minute period of circulatory arrest to reconstruct the distal aorta after iatrogenic injury during surgery. The third patient suffered intraoperative air embolism during LVAD implantation. Peripheral thrombotic complications involved one patient who developed an isolated lower extremity arterial thrombus, which was successfully treated via surgical thrombectomy and a second patient who suffered from an uncomplicated lower extremity deep venous thrombosis ten days after rFVIIa administration. Both of these patients were discharged from the hospital without further complication.

## Discussion

rFVIIa is a potent clotting factor that is currently FDA-approved only for the treatment of severe bleeding episodes in hemophiliacs with factor inhibitors or patients with FVII deficiency, although numerous reports are now appearing in the literature describing its off-label perioperative use in the management of diffuse hemorrhage not amenable to surgical control. The precise mechanism by which rFVIIa works is not completely clear, however *in vitro *studies investigating the mechanism of action of rFVIIa postulate that it involves generation of thrombin by initial binding to tissue factor and subsequent activation of factor X (FXa) on the platelet surface (i.e. a phospholipid surface); FXa in combination with activated factor V (i.e. prothrombinase complex) leads to thrombin formation. This occurs in the absence of factor VIII or factor IX. [19-21]. The extent of thrombin activation relates to the concentration of activated factor VII achieved. Partial thrombin generation occurs at rFVIIa concentrations approximating 50 nM, whereas full activation of thrombin, referred to as a "thrombin burst", is achieved with higher levels (100 to 150 nM)[22]. This pronounced thrombin activity on thrombin-activated platelet surfaces leads to a stabilized thrombin plug and tight fibrin structure resistant to lysis [23]. This experimental evidence supports the concept that rFVIIa is potentially safe with minimal thrombo-embolic complication because it is effective at sites of vascular injury where there is localized expression of tissue factor and activated platelets. However, the thrombotic risks associated with rFVIIa may be theoretically increased in clinical situations when there is systemic (e.g. disseminated intravascular coagulation) or localized (atherosclerotic plaque) pathologic expression of tissue factor, and or circulating activated platelet microparticles [24].

rFVIIa has been shown to be highly effective in correcting coagulopathy in patients with clotting factor deficiencies and/or neutralizing antibodies to coagulation cascade proteins. In such a population, as of 2003, several thousand patients had received over 700,000 doses with complications reported in only 1–2% of cases[25,26] The most frequently encountered complications included thromboembolic events, myocardial infarction, and disseminated intravascular coagulation, although any direct relationship between the administration of rFVIIa and the latter two adverse events is vague at best due to predisposing or pre-existing factors in the affected patients[27,28] Currently, it is unclear whether rFVIIa is safe and can effectively correct acquired coagulopathy in critically ill cardiac and thoracic surgery patients without the increasing the risk of thromboembolic complications[28,29]

To date, six randomized, placebo-controlled, double-blind studies including all surgical patients have been published, with two of these coming from the same group[4,30-33] Two of these studies report significant decreases in both the number of patients requiring massive (>10 units PRBC) transfusion, and in the total units of transfused blood [4,32]. Two studies showed a non-significant trend toward similar results [32,33], and the last two showed no significant differences between the treatment and placebo groups [30,31]. One of the trials that did show significant reduction in transfusion requirements involved only cardiac surgical patients, but none of the subjects underwent transplant or VAD implantation/explantation[4]. None of the six trials showed any significant increase in the number of adverse events in patients receiving rFVIIa. However, recent studies have demonstrated an increased incidence of thrombo-embolic complications associated with rFVIIa administration[34,35].

VAD insertion is a complex surgical procedure with perioperative bleeding occurring in nearly half the cases [1], and also associated with long term thromboembolic complications based on the type of the implant used[2]. The current literature addressing the use of off-label rFVIIa in cardiac surgery patients consists of isolated case reports of only a single subject at a given institution[13-15,28,29,36-45] has been reviewed by Despotis et al[46] Eight such studies deal with cardiac transplantation and/or VAD placement [47-54] Most of these single case reports indicate that rFVIIa administration resulted in significant decrease in bleeding without any thromboembolic events. One patient developed fatal, diffuse intravascular thrombosis during administration of FEIBA after receiving two doses of rFVIIa several hours prior[29] Of the studies involving multiple patients with rFVIIa administration during or after cardiac surgery; [13-15], [55-66], five address those undergoing OHT (eight patients total) or VAD/ECMO placement (14 patients total). In four of the five studies there were some thromboembolic adverse events occurring after rFVIIa administration [57,58,60,61]

In our evaluation of 17 patients undergoing OHT and/or VAD placement, we are able to demonstrate a significant improvement in virtually every measure of effective hemostasis following administration of between 24 and 189 μg/kg of rFVIIa. Most importantly, these parameters that showed improvement included six and twelve hour blood loss, units of blood transfused, and PT/PTT values. This is consistent with a recent review of the literature for use of rFVIIa following cardiac surgery[46] Unfortunately, despite such dramatic improvement in clinical values, our patient population still had an overall 29% (5/17) mortality rate, with two of those deaths occurring within 24 hours of receiving the treatment. One patient died from acute allograft failure that failed to recover with ECMO support. Another patient died due to multiorgan failure following an emergency VAD placement. Three patients died secondary to massive cerebrovascular accidents. Although there were pre-existing clinical features increasing the risk of thrombosis in 3/5 patients, rFVIIa was administered in all the cases only after at least two rounds of conventional hemostatic therapy failed to stop the bleeding. This acute mortality is likely reflective not only of the critically ill nature of our patients in general, but also the very large volume of transfused blood products, and associated coagulopathy, prior to administration of rFVIIa. The limited finding in this subset of our patients is consistent with the findings of other studies that had used rFVIIa when conventional hemostatic therapy failed to stop hemorrhage [67,68]. Of the three patients that died of massive strokes, two were confirmed to be embolic although one was clearly related to HIT and the other had significant risk factors for atheroembolism.

Two patients had peripheral thrombotic complications. The patient that developed a lower extremity thrombus had LVAD implantation along with aortic root homograft following an aortic valve redo operation with an aortic root abscess secondary to bacterial vegetations on the aortic valve. Another patient had DVT ten days after rFVIIa administration. His post-op course was complicated by a low cardiac index requiring intra-aortic balloon, episodes of atrial fibrillation and acute renal failure requiring hemodialysis before developing DVT.

All these patients had other reasons for the thromboembolic events however, significant thromboembolic complications could certainly be a result of the administration of rFVIIa, and they speak strongly to the necessity of further controlled studies to evaluate the drug in the setting of these complex, critically ill patients.

A summary report of adverse events (AE) including thromboembolic events following administration of rFVIIa to FDA adverse event reporting system (AERS 1999–2005) was recently published[34] There were 599 AE during this time period of which 220 were thromboembolic, in more than 10,000 patients (patient exposure based on a commercially-derived estimate, Premier Healthcare Informatics, Premier Inc, Charlotte, NC). A vast majority of this thromboeombolic events occurred following off-label use of rFVIIa. Of the 220 thromboembolic AE, there were 67 mortalities (30%) and 67% (45/67) of these deaths were thought to be related to the thromboembolic complication. The frequency of the thromboembolic AE cannot be determined from this data, since the reporting to AERS is purely voluntary and many AE are not reported.

Karkouti et al in their study comparing the outcomes of cardiac surgery patients that received rFVIIa for refractory bleeding to propensity matched controls from general cardiac surgery patients found that the mortality risks in both the groups was comparable, with higher risk of renal failure and stroke in the group receiving rFVIIa[57,61]. Recently, the same group in their observational study compared the unadjusted and risk-adjusted adverse events in cardiac surgery patients with refractory bleeding that received or did not receive rFVIIa. They found that when no adjustment was made, rFVIIa administration was strongly associated with composite adverse events (OR 2.41; 95% CI 1.58–3.67). However, after risk adjustment using propensity matching for other confounding factors like red cell transfusion, CPB time, they found that rFVIIa administration was no longer associated with composite adverse events (OR 1.04; 95% CI 0.60–1.81)[58]

Although the timing of rFVIIa administration was not strictly defined in our study, some have suggested that rFVIIa would be better used earlier in the course of significant postoperative hemorrhage or after a dictated number of transfusion units[32,58,67,69]

Dosing of rFVIIa for off-label settings is not standardized and is evolving based partly on cost consideration and also evidence that doses lower than the recommended dose for hemophiliacs (i.e. 90 ug/kg) may be effective in such settings. A recent study found that a median rFVIIa dose of 17 ug/kg (Range: 11.1 ug/kg to 21.7 ug/kg) showed satisfactory results in cardiac patients with intractable bleeding [70]. Yet another study from Netherlands found that rFVIIa dose of 40 ug/kg stopped bleeding in seven patients who had uncontrolled bleeding after cardiac surgery[71]. In our group, patients were administered an average dose of 40 μg/kg at the first and the second instance. A third dose at an average of 31 μg/kg was administered to 4/15 patients. The amount of rFVIIa used was based on the patient's clinical condition in consultation with the surgeon, the anesthesiologist and the transfusion medicine team. We have previously reported a catastrophic ECMO thrombosis in a patient who had received rFVIIa along with prothrombin complex [29]. Based on the concerns for thrombotic complications, patients are usually started with a lower dose of rFVIIa and based on previously published guidelines[18]. Patients that showed improvement after administration of one dose of rFVIIa but continued to bleed may receive additional doses of rFVIIa after receiving additional conventional blood therapy. No patients received more than three doses of rFVIIa.

## Conclusion

In our retrospective review of patients undergoing VAD implantation or removal and OHT, we found that rFVIIa appeared to be effective in reducing life-threatening bleeding refractory to conventional hemostatic blood component therapy. We also found that administration of rFVIIa may be associated with significant decrease in blood loss and blood component use. Nearly a third of our patients had thromboembolic events, of which three were fatal. Considering that this patients received rFVIIa after at least two rounds of conventional hemostatic therapy failed to stop bleeding with other predisposing clinical conditions including life-threatening bleeding (i.e., with hyoperfusion and target organ injury) that would predispose them to develop thromboembolic complications and a higher mortality rate, a clear association with rFVIIa administration cannot be made. Our data thus adds to the growing literature regarding the use of rFVIIa in treatment of refractory coagulopathy associated with non-coronary complex cardiac surgeries. However, additional randomized investigations to study the optimal timing, dosing and associated thromboembolic risks of rFVIIa are needed. In summary it can be concluded that rFVIIa administration can be helpful in the treatment of patients with life-threatening bleeding following complex non coronary cardiac surgery.
